# Assessing the validity of an OSCE developed to assess rare, emergent or complex clinical conditions in endocrinology & metabolism

**DOI:** 10.1186/s12909-021-02653-4

**Published:** 2021-05-20

**Authors:** Stephanie Dizon, Janine C Malcolm, Jan-Joost Rethans, Debra Pugh

**Affiliations:** 1 Department of Medicine, The Ottawa Hospital, 1967 Riverside Drive, 4th Floor, Ontario K1H 7W9 Ottawa, Canada; 2 Division of Endocrinology & Metabolism, The Ottawa Hospital, 1967 Riverside Drive, 4th Floor, Ontario K1H 7W9 Ottawa, Canada; 3 Faculty of Medicine, University of Ottawa, Ottawa, Ontario Canada; 4 Faculty of Health, Medicine and Life Sciences, Maastricht University, Maastricht, Netherlands; 5 Division of General Internal Medicine, The Ottawa Hospital, Ottawa, Ontario Canada; 6 Medical Council of Canada, Ottawa, Ontario Canada

**Keywords:** OSCE, Kane’s validity framework, Endocrinology, Competency-Based Medical Education

## Abstract

**Background:**

Assessment of emergent, rare or complex medical conditions in Endocrinology and Metabolism (E&M) is an integral component of training. However, data is lacking on how this could be best achieved. The purpose of this study was to develop and administer an Objective Structured Clinical Examination (OSCE) for E&M residents, and to gather validity evidence for its use.

**Methods:**

A needs assessment survey was distributed to all Canadian E&M Program Directors and recent graduates to determine which topics to include in the OSCE. The top 5 topics were selected using a modified Delphi technique. OSCE cases based on these topics were subsequently developed. Five E&M residents (PGY4-5) and five junior Internal Medicine (IM) residents participated in the OSCE. Performance of E&M and IM residents was compared and results were analyzed using a Generalizability study. Examiners and candidates completed a survey following the OSCE to evaluate their experiences.

**Results:**

The mean score of IM and E&M residents was 41.7 and 69.3 % (*p* < 0.001), respectively, with a large effect size (partial η^2^ = 0.75). Overall reliability of the OSCE was 0.74. Standard setting using a borderline regression method resulted in a pass rate of 100 % of E&M residents and 0 % of IM residents. All residents felt the OSCE had high value for learning as a formative exam.

**Conclusions:**

The E&M OSCE is a feasible method for assessing emergent, rare and complex medical conditions and this study provides validity evidence to support its use in a competency-based curriculum.

## Background

The shift towards competency-based medical education (CBME) in post-graduate medical education requires frequent assessment of physician competencies across various clinical contexts within each specialty [1]. However, there is significant variability in the cases that residents may encounter during their training. Because of this, residents may never be assessed on their ability to manage some rare, emergent or complex conditions which would be essential for becoming an expert in their field. If residents have had limited or no exposure to these cases, then their ability to manage patients with these conditions may negatively impact future patient outcomes. This leads to the question of how do we evaluate competencies in certain clinical scenarios that are not easy to access in the learning environment? Ideally, one would like to observe how trainees perform during real clinical encounters, however exposure to some conditions may be limited and so other assessment opportunities must be sought. In these cases, simulation in the form of Objective Structured Clinical Examinations (OSCEs) may be useful [2].

In a CBME model, it is important to demonstrate progression of clinical skills through frequent observational assessments. Within a programme of assessment, multiple methods of assessment can be combined to achieve an overall impression of competency within a specific domain [3]. Part of this design will involve an increasing number of workplace assessments to assess trainees’ progression along a continuum towards expertise. This is possible in situations where a clinical problem is common, with several opportunities for hands-on advancement of skills. However, in clinical scenarios that are rare, emergent or complex, assessing competence in a real-life setting may not be consistently possible during their training period.

Introducing a formative OSCE to address progressive assessment across training years may be of benefit in a CBME model where assessment data from multiple methods contribute to competency decisions [3]. Furthermore, the use of OSCEs as progress tests have been shown to be useful in discriminating between levels of training within an Internal Medicine (IM) program [4], but has not been described in Endocrinology programs. Although it is known that OSCEs are resource-intensive, they can be viewed as a more suitable method for assessing cases that are neither easily accessible in the workplace (i.e., rare, emergent, or complex) nor easily assessed in a written format [2]. OSCEs are purported to be objective and structured but they are not necessarily considered superior to other methods, rather, they can be complementary within a programme of assessment [5]. However, when deciding to include a particular assessment into a curriculum one must consider sources of validity evidence to justify its position in the program [6]. An assessment with robust validity evidence enables assessors to trust that the scores obtained represent the construct it intends to measure [7].

Messick and Kane’s modern validity frameworks aim to gather evidence from various sources in order to demonstrate *construct validity* (i.e., the degree to which the test measures what it purports to measure) [8–11]. However, the advantage of Kane’s validity framework is that it prioritizes validity evidence in key phases or inferences within the validity argument: *scoring, generalization*, *extrapolation* and *implications* [12, 13]. The first step of *Scoring*, seeks to ensure that the scores obtained from the observed actions best represent the performance [8]. The second step of *Generalization* refers to obtaining an overall test score that represents the general performance test setting in equivalent types of tests [8, 12]. Thirdly, the *Extrapolation* phase aims to determine if the observed performance correlates with real world performance or other measures of the same or similar performance domains [14, 15]. Lastly, the *Implications* of the assessment tool includes decision making (i.e., pass/fail) or consequences of the test on those assessed [14]. To date, there have been no published articles on assessing validity evidence in OSCEs within the Endocrinology & Metabolism (E&M) specialty.

The purpose of this study is two-fold: (1) to develop a pilot OSCE for E&M residents to assess their management of rare, emergent or complex E&M scenarios that may be missed in clinical training; and (2) to gather validity evidence for this OSCE in light of Kane’s framework. We aim to address the following questions: To what extent does the validity evidence support the use of the E&M OSCE as a formative assessment for rare, emergent or complex cases? More specifically, to what degree does the OSCE represent the constructs it intends to measure? Finally, what is the perceived value for learning from a resident’s perspective? In order to achieve this, we carefully designed an OSCE that represents what we intended to assess, while collecting validity evidence.

## Methods

### OSCE Development and Design

#### Needs Assessment

An electronic survey (via Survey Monkey©) was sent to all 13 E&M Program Directors across Canada as well as 29 recent E&M graduates (i.e., those who graduated within the last two years) to seek their opinion regarding gaps in their residency training program and which topics they believe would be important to consider for an OSCE.

A list of rare and emergent cases was included in the survey to rank (determined from objectives of E&M training and content expert agreement), in addition to a free-text area to suggest topics. Consensus was ascertained using a modified Delphi technique, involving two rounds of ranking the top ranked priority topics. From this, a list of five top-ranked topics was identified, all of which are reflected in the Royal College of Physicians & Surgeons of Canada (RCPSC) Objectives for Training for E&M residents (http://www.royalcollege.ca/rcsite/ibd-search-e?N=10000033+10000034+4294967098).

#### Case Development

Five cases based on the top-ranked were developed by a specialist in E&M and were reviewed by three additional content experts. Through an iterative process, each case was reviewed and revised by three of the study investigators (SD, JM and DP) and the current E&M Program Director at the University of Ottawa (UofO).

#### Setting and administration

The OSCE was administered at the UofO in the 2018–2019 academic year. To accommodate all candidates, the OSCE was administered twice, using one track (5 cases consecutively). Five candidates participated in each administration (total *n* = 10). Each administration contained the same five cases and each case lasted 12 min. Each candidate was assessed by a unique rater for each station and the raters remained unchanged for each administration.

#### Context and subjects

Five E&M resident physicians [3 PGY (Post Graduate Year)-4 s and 2 PGY-5 s] were recruited as participants. Additionally, five Internal Medicine (IM) residents (PGY1 to 3) were recruited as a comparison group. All residents went through an informed consent process with the Research Assistant. Immediately preceding the OSCE, each resident group participated in an orientation session (led by SD) to explain the purpose and structure of the OSCE and to address any concerns.

#### Examiners (raters)

Raters included faculty experts (four Endocrinologists and one Internal Medicine Specialist). An orientation session prior to the OSCE was provided to the examiners (led by DP) to explain the purpose and structure of the OSCE, to ensure that they were familiar with the use of scoring instruments, and to provide the opportunity to ask questions about the OSCE.

#### Standardized patients

Experienced standardized patients (SPs) were recruited and received training for their roles by experienced trainers, in line with current global standards of SP training [16].

#### Scoring Instruments

Participants were assessed by raters using scenario-specific scoring sheets (consisting of checklists and a series of rating scales) with items that were case-specific. Each contained “key feature” items that are deemed to be important actions necessary to meet the topic objectives. The case-specific checklists were developed by the principal investigator (SD) and reviewed using consensus agreement amongst content experts, including the E&M Program Director. Rating scales were used to rate performance in the areas of: (1) *Organizational skills*; (2) *Ability to communicate plan*; and (3) *Ability to prioritize acute medical issues*. A global rating score (GRS) designed to rate candidates’ overall competence was also included.

#### Analyses

Using Kane’s modern validity framework, sources of validity evidence were gathered and analyzed in the domains of *Scoring, Generalization, Extrapolation*, and *Implications*.

#### Scoring

Weighting for the checklist and rating scale components was determined by a panel of experts in E&M and OSCE administration. A total score for each case was derived by combining the total checklist scores (70 %) with the rating scales (30 %). Descriptive statistics were calculated, as well as item-total correlations for each case using SPSS Software version 25. To ensure the integrity of data, quality assurance measures were employed during data collection and data entry. Immediately following the OSCE, the examination staff ensured that all checklists and rating scales were completed accurately. Data entry was double checked by experienced staff who employed quality control checks to ensure accuracy of scores entered in analyses.

#### Generalizability

Although this was a small-scale OSCE, the blueprint was derived using consensus methods to gain input from various stakeholders.

Measures of Generalizability include the reliability (i.e., reproducibility) of the scores, and the degree to which the stations represent the domain of interest. Since stations have multiple factors that can contribute to variance, Generalizability Theory (G-theory) was applied to quantify to what degree each variable (i.e., resident type, training level, participants, or stations) contributed to the overall variability in the scores. To generate the variance components, a mixed analysis of variance was conducted with students nested with discipline and crossed with stations. These variance components were then used to generate the reliability of the exam scores. Because we were interested in scores and not the reliability of the pass/fail standard, a relative reliability was used. We also used the results of the generalizability analysis to conduct a decision study, which uses the variance components to derive estimates of reliability if various factors in the model are varied. This analysis will be useful for determining how many stations are needed to produce a reliable set of exam scores.

#### Extrapolation

The ability of the OSCE to discriminate between novice (PGY 1–3) and expert groups (PGY 4–5), was measured using an independent *t* test.

#### Implications

Although this was designed as a formative examination, the Borderline Regression Method (BRM) was used to demonstrate how to apply methods for standard setting. This method involves a linear regression approach where all candidates’ checklist scores are regressed onto their global rating score to produce a linear equation [17]. The cut-score is determined by inserting the midpoint of the GRS (which is 3.5 on the current 6-point scale) into the equation, which results in a corresponding predicted checklist score [17].

Pass-fail decisions on this OCSE had no bearing on participants’ progression through the E&M program and were used to help determine if certain stations were unfairly difficult or if there were areas of underperformance that would require attention. Identification of difficult stations were utilized to inform curriculum change to promote learning in weaker areas.

To obtain the residents’ perspective, a post-OSCE survey was used to evaluate the degree of acceptability of the examination, and the degree to which they felt the OSCE has value for learning.

## Results

### Needs Assessment Survey

**Fig. 1:**
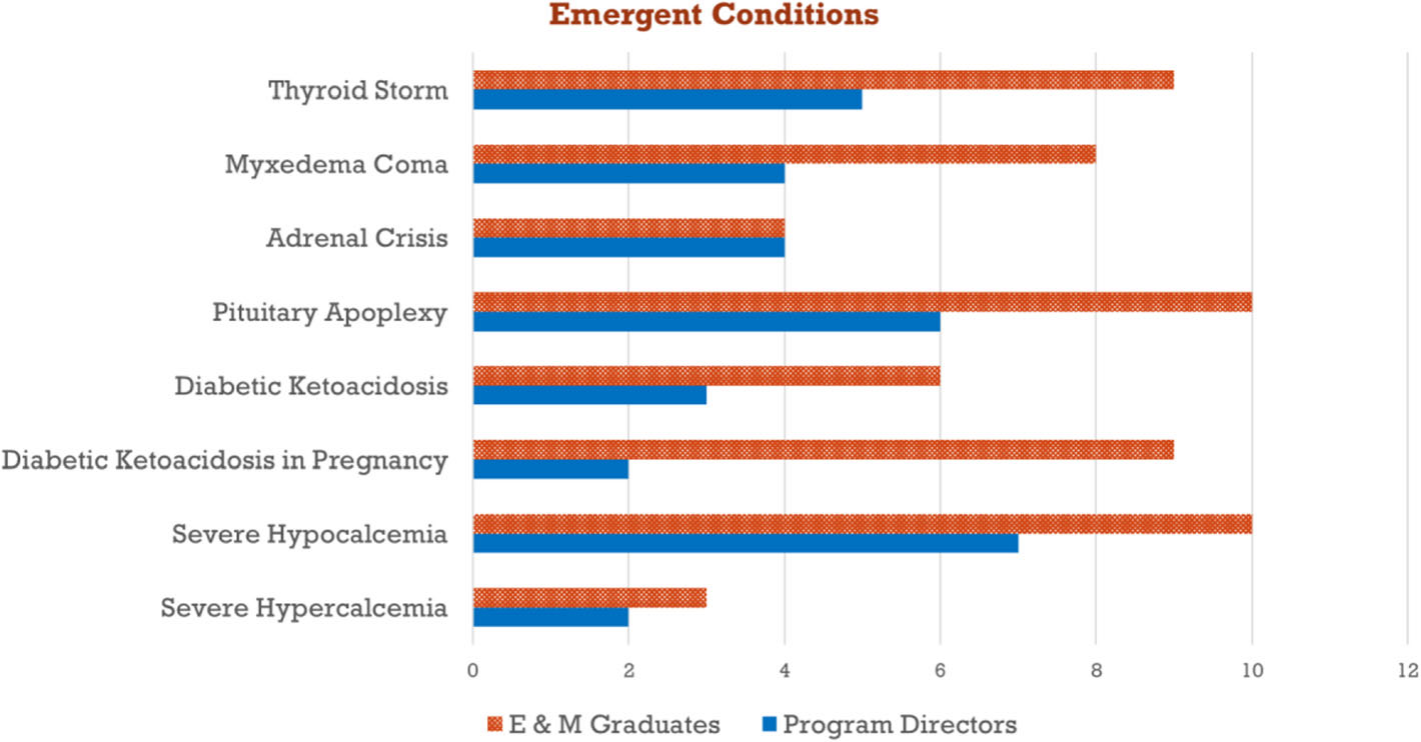
Frequency of “Emergent Conditions” selected in survey #1

Seven out of 13 PDs (54 %) and 14/29 (48 %) E&M Graduates responded to the initial survey, with an overall response rate of 50 % (21/42). The top five selected topics from the “emergent” category in order of frequency were: (1) thyroid storm, (2) pituitary apoplexy, (3) severe hypocalcemia, (4) myxedema coma and (5) diabetic ketoacidosis in pregnancy (Fig. 1).

**Fig. 2:**
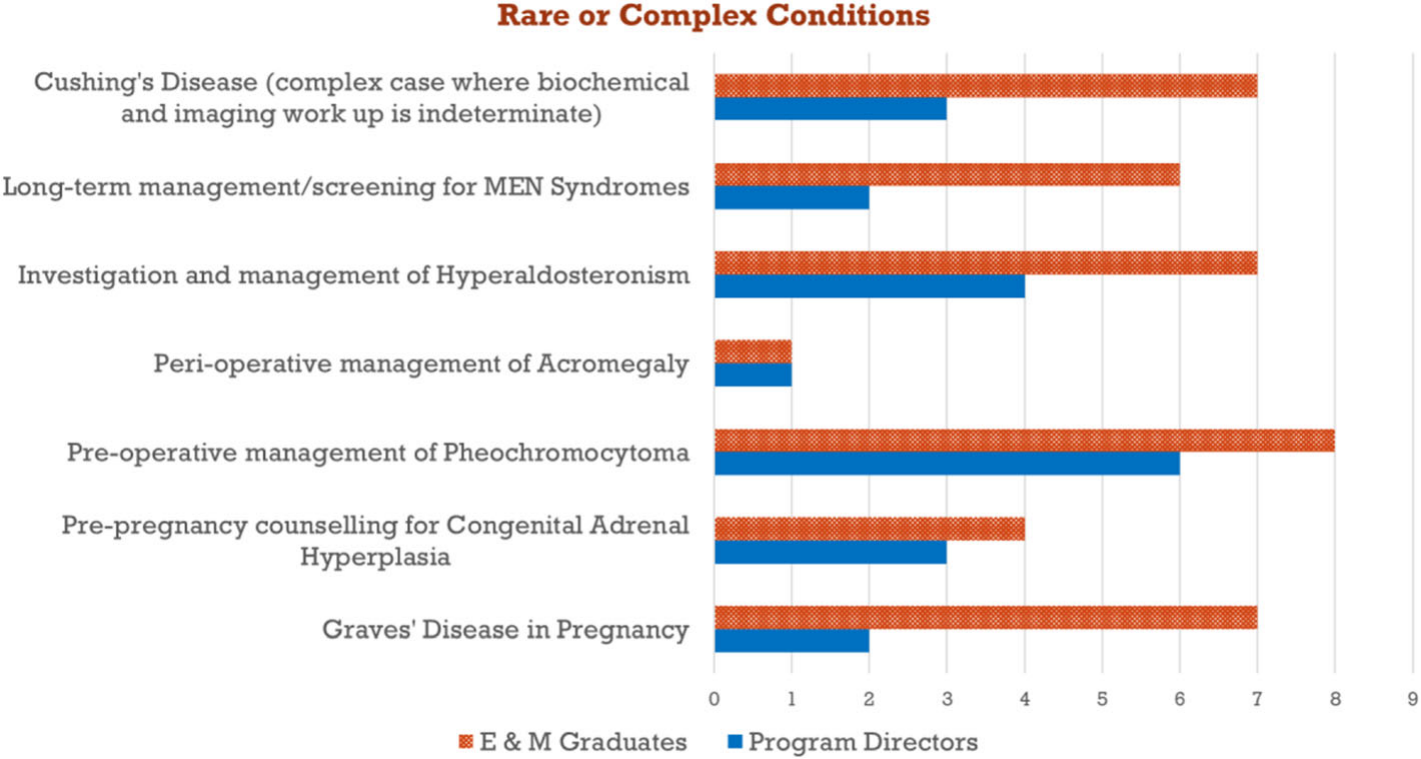
Frequency of “Rare or Complex Conditions” selected in survey #1

The top five selected topics in the “rare or complex” category were: (1) complex Cushing’s disease, (2) investigation and management of hyperaldosteronism, (3) pre-op management of pheochromocytoma, (4) Graves’ disease in pregnancy and (5) MEN syndromes (Fig. 2). The total frequencies per topic were totalled and the top 10 topics were subsequently used for ranking in the second survey.

**Fig. 3:**
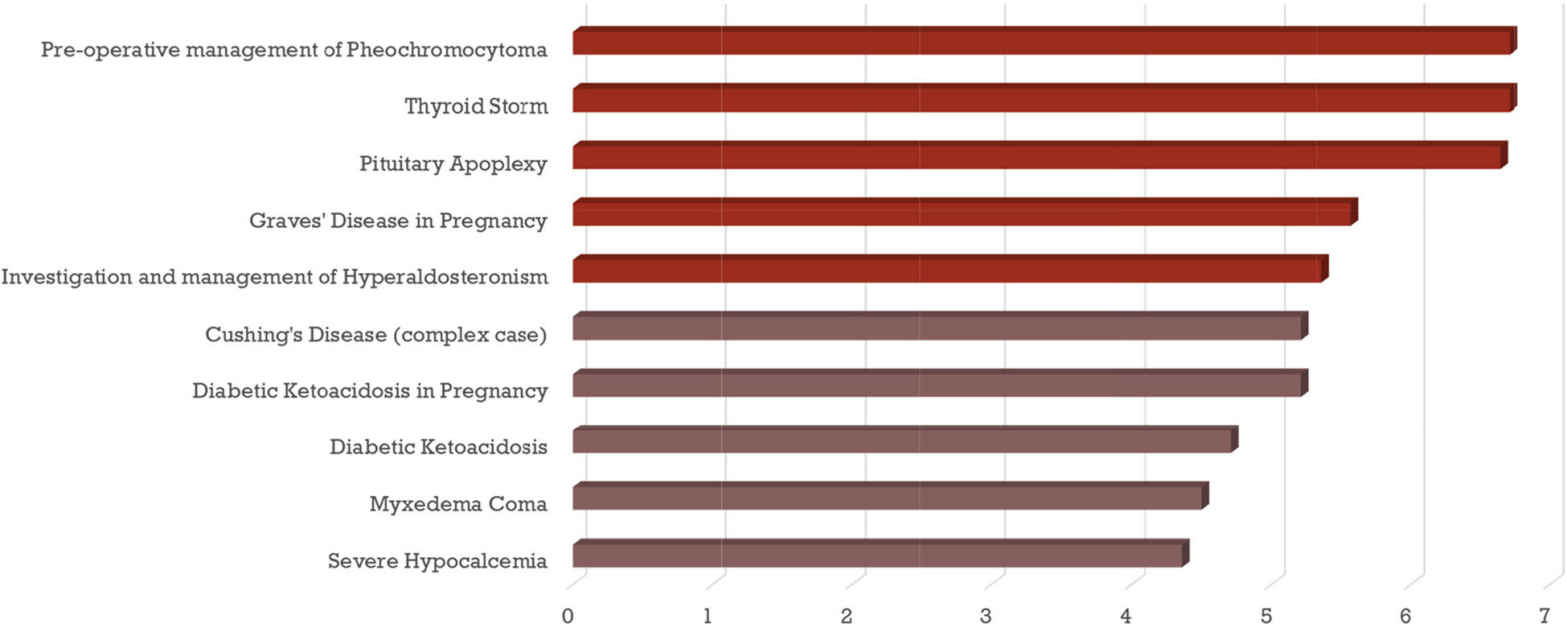
Top 10 Ranked Cases – Results from survey #2

There were 14 respondents from the second survey (6 PDs and 8 E&M Graduates) of the original 42 that were invited (33 % response rate). Five topics emerged from the ranking exercise in the second survey: (1) pre-operative management of pheochromocytoma; (2) thyroid storm; (3) pituitary apoplexy; (4) Graves’ disease in pregnancy and (5) investigation and management of hyperaldosteronism (Fig. 3). These topics were used as the basis for OSCE case development.

 To ensure that the top ranked cases adequately represented the construct we intended to measure, the study investigators (SD, JM, DP) reviewed the results in detail to come to a consensus and were deemed suitable to meet the objectives of this OSCE.

### Evidence for validity

#### Scoring

Mean scores out of 10 and item-total correlations (ITCs) per station are represented in Table 1. Stations scores with ITCs over 0.3 are considered to have good correlations with the total score. The station ITCs are quite high, indicating that scores for all stations are highly correlated with the total score for the entire OSCE. This is not surprising, given that the OSCE was designed to assess a relatively focused area of clinical practice (i.e., rare, emergent and complex conditions in E&M).

**Table 1:** Mean Scores and Item-Total Correlations (ITC)

	Mean	Range	Standard Deviation	N	ITC	
Stn 1. Thyroid Storm	6.410	4.0–9.0	1.5567	10	0.90	
Stn 2. Pituitary Apoplexy	6.760	4.1–8.5	1.7411	10	0.66	
Stn 3. Pheochromocytoma	5.190	1.8–8.7	2.1997	10	0.85	
Stn 4. Graves’ in Pregnancy	4.820	1.7–7.3	1.9871	10	0.93	
Stn 5. Primary Hyperaldosteronism	4.580	2.2–7.7	2.0060	10	0.77	

#### Generalizability

The results of the generalizability analysis are displayed in Table 2. The discipline (d) effect (Endocrinology & Metabolism versus Internal Medicine) accounts for 59 % of the variance of scores, indicating a difference between E&M residents and IM resident performance. Participants within each discipline (p:d) accounted for 10 % of the variance, indicating that there were small differences between people within each discipline. The differences between stations accounted for 14 % of the variance, however the interaction between stations and discipline (ds) was 0 %, suggesting that the stations performed similarly for each discipline.

**Table 2:** Generalizability Analysis

facet	variance	% variance	Description
d	3.66	59	Variance due to differences between disciplines
p:d	0.60	10	Variance due to difference between people within a discipline
s	0.87	14	Variance due to differences between stations
ds	0	0	Variance due to stations as a function of discipline
ps:d	1.05	17	Variance due to people within a discipline getting different scores on the stations plus random error

*d* discipline, *p* people, *s* stations

The G-coefficient, considering both disciplines and stations simultaneously, was 0.74, which is sufficient for lower-stakes exams [18]. If this was designed to be a higher stakes examination, 7 stations in total would be needed to achieve a reliability of 0.80.

#### Extrapolation

The mean score in group 1 (E&M Residents) was 69.3 % for the entire OSCE, compared to 41.7 % for group 2 (IM group) (Table 3). Independent *t* tests for group 1 versus group 2 show a significant difference between the two groups (*p* = 0.001) (Table 4).

**Table 3:** Comparison of total OSCE scores between groups, Internal Medicine (PGY1-3) and Endocrinology & Metabolism (PGY4-5)

Group	Mean	Std. Deviation	N
1	69.320	9.3900	5
2	41.720	8.6146	5
Total	55.520	16.8455	10

**Table 4:** Independent Samples T-Test

					95 % CI for Cohen’s d	
	t	df	p	Cohen’s d	Lower	Upper
Total	4.843	8.000	0.001	3.063	1.101	4.951

*Note.*  Student’s t-test

Differences in performance based on discipline were calculated using a factorial ANOVA with discipline as a between subjects factor and the total score as the dependent measure. There was a statistically significant difference between IM and E&M Residents: F (1,8) = 23.46, (*p* = 0.001), with a very large effect size (partial eta^2^ = 0.75).

### Implications

#### Pass-fail results

Using the BRM, the overall OSCE cut score was determined by totaling the cut scores for all 5 stations, resulting in a total cut score of 29.6 out of 50 (equivalent to 59.2/100). Residents in the E&M Group passed more stations than the IM Group (Table 5). All 5/5 (100 %) of E&M Residents would have passed the entire OSCE, conversely, 0 % of IM Residents would have passed if this was considered high stakes, which is not surprising given that the was an OSCE designed to assess rare, emergent and complex conditions in E&M.

**Table 5:** Pass-Fail Station Scores per Candidate

		Stn 1	Stn P/F	Stn 2	Stn P/F	Stn 3	Stn P/F	Stn 4	Stn P/F	Stn 5	Stn P/F	Total /100	PASS / FAIL
*Discipline (year)*	*Mean*	*6.4*		*6.8*		*5.2*		*4.8*		*4.6*		*55.5*	
	***Cut***	***6.4***		***6.1***		***5.2***		***7.4***		***4.5***		**59.2**	
EM (PGY4)		5.8	F	7.8	P	5.1	F	6.3	F	5.0	P	60.0	PASS
EM (PGY4)		5.8	F	7.2	P	6.2	P	5.2	F	5.3	P	59.4	PASS
EM (PGY4)		8.0	P	8.5	P	5.5	P	6.8	F	7.2	P	72.0	PASS
EM (PGY5)		9.0	P	8.3	P	8.7	P	6.8	F	7.7	P	81.0	PASS
EM (PGY5)		8.2	P	8.3	P	7.8	P	7.3	F	5.5	P	74.2	PASS
IM (PGY1)		4.8	F	5.4	F	1.9	F	2.2	F	2.2	F	33.0	FAIL
IM (PGY1)		4.0	F	5.3	F	1.8	F	1.7	F	3.1	F	31.8	FAIL
IM (PGY2)		6.3	F	8.2	P	4.5	F	3.7	F	2.3	F	50.0	FAIL
IM (PGY2)		6.0	F	4.1	F	4.7	F	3.7	F	5.1	P	47.2	FAIL
IM (PGY3)		6.2	F	4.5	F	5.7	P	4.5	F	2.4	F	46.6	FAIL

#### Post OSCE Survey results

##### Resident Experience

The majority of IM residents had not encountered any of these OSCE scenarios in real life. However, the E&M residents reported variable exposures to these cases in the clinical world. Overall, residents felt that the OSCE cases were relevant to their learning with 57 % stating that the topics *“mostly aligned with their learning objectives*” and 43 % stating that they *“completely aligned with their learning objectives*”. Further to this 57 % felt that the OSCE was “*likely to drive further learning*”, and 43 % stated it was “*very likely to drive further learning*”.

##### Examiner Experience

All five examiners completed the post-OSCE survey, reporting that their case was either a “good” representation (80 %) or an “excellent” representation (20 %) of a true patient encounter. Furthermore, most rated the SP performances as “excellent” (80 %), while the remainder rated it as “good” (20 %) for their respective stations.

## Discussion

Because many challenging clinical conditions may not be encountered during a resident’s E&M training, we developed and administered a novel OSCE to assess their knowledge and skills in managing rare, emergent and complex conditions. Validity evidence for this OSCE was gathered and analyzed through the lens of Kane’s validity framework. Evidence for ‘scoring’ was demonstrated through the use of multiple content experts in the development of the rating instruments, through the careful training of raters and SPs, as well as through the high item total correlations that were found. ‘Generalization’ evidence included rigorous methods to determine the exam content (i.e., national survey, consensus methods, and expert review) and relatively high exam reliability as determined by a G study. The differences demonstrated between experts and novices provided evidence for ‘extrapolation’. In addition, the survey data confirmed that raters and trainees viewed the experience as authentic and relevant. And finally, although this was a formative OSCE, evidence for ‘implications’ was demonstrated through the use of standard-setting methods and by informing curricular changes.

Current literature supports the value of simulation in procedural based specialties such as Surgery, Anesthesia, and Emergency Medicine for both training and assessment [19, 20]. An OSCE was developed to assess resuscitation skills in Emergency Medicine, showing the value of simulation in acute and emergent settings, by differentiating between skill levels [19]. Similarly, a simulation curriculum called the National Anesthesiology Simulation Curriculum (CanNASC), has been implemented across 17 Anaesthesia training programs in Canada designed to assess knowledge or skills gaps in the curriculum [21]. In contrast, there is a lack of data on the use of simulation in medicine sub-specialties. Our study is the first to describe the development and evaluation of an OSCE for rare, emergent and complex conditions in E&M. Since some E&M conditions are encountered so infrequently, certain topics may be missed during the course of a resident’s sub-specialty training. OSCEs therefore could have a role in providing an opportunity to practice in an environment that does not impact patient safety [22]. Although our OSCE was designed to specifically assess difficult areas of assessment in the E&M specialty, similar methods could be used to produce an assessment tool in any clinical context.

The OSCE was intended to represent a sample of priority topics felt to be rare, emergent or complex in the E&M specialty. At present, there have been no established methods for assessing E&M Residents for this category of conditions within the educational literature. The Royal College of Physicians and Surgeons of Canada require that E&M residents meet objectives including managing Endocrine emergencies, in addition to other rare conditions captured within this OSCE [23]. However, since there is no ‘practical examination’ for E&M board certification, residents may never be assessed in this domain. It would be important that E&M residents are exposed to these important clinical topics both to drive learning and provide an opportunity for competency assessment. As we progress towards CBME, E&M programs will require more collection of data to support competency decisions, particularly through observation of trainees. Assessment of competencies in the form of Entrustable Professional Activities (EPA) require progressive levels of observation [3]. The OSCE is a useful tool for providing opportunities for observation in circumstances where clinical scenarios are rarely encountered, therefore providing an opportunity for assessment in areas that may be missed in the clinical environment. As EPAs have become the main unit of competency assessment in post-graduate training programs, it would be important to ensure that future OSCE scores and rating scales align with milestones to inform EPAs.

As evidenced by the positive learning experience comments from the residents, having a formative OSCE in this category of topics, provides an opportunity to drive further learning. It is known that competence is “context specific” and can vary based on the situation, the learner and assessor [24]. However, within a programme of assessment, this OSCE can serve as one of many different instruments used to provide data towards the continuum of competency assessment.

Although it is recognized that it may not be possible to assess every competency in a CBME curriculum [3], providing a sample of potentially missed scenarios as simulated cases can serve as an impetus towards “assessment for learning”. Including simulated cases within a programme of assessment, may allow residents to use feedback as a vehicle to re-focus their learning to fill competency gaps [5]. Pugh et al. studied how formative OSCEs affect learning by surveying Internal Medicine residents, illustrating that residents view OSCEs as “a hurdle to overcome”, while simultaneously viewing it as a platform to receive feedback and learn [25]. The residents in our OSCE mirrored this belief that having exposure to these cases was likely to drive further learning. If this type of assessment is implemented into an E&M training program, residents may be more self-aware of their learning gaps and will hopefully mitigate these gaps before they enter independent practice.

### Limitations

Potential limitations of this study include the limited sample size and the implementation of the OSCE at a single centre. If additional funding was available, recruitment of additional E&M residents outside of our institution would be beneficial to increase generalizability outside of our local context. We would have ideally administered the OSCE for E&M residents from multiple programs and would seek to do this at a national level, for example, at an annual conference. Although not all possible topics could be captured in this OSCE, the performance may be representative of resident performance on topics within this domain based on their similarity in cognitive complexity and level of expertise.

Although we were able to differentiate between the junior IM group (PGY 1–3) group and E&M group (PGY 4–5) based on performance in this OSCE, we would want to further delineate performance between PGY-4 vs. PGY-5 vs. practising physician for this specialty exam. This would require a larger scale study with more resident participants in each PGY group if we intend to see a difference between years of training. Administering the OSCE annually using different cases within a similar clinical context would provide data to observe progression in achieving competencies.

## Conclusions

The development and evaluation of a pilot OSCE designed for assessing rare, emergent and complex topics in Endocrinology & Metabolism was shown to be a feasible means to distinguish between levels of expertise. We applied Kane’s framework to acquire validity evidence and we can infer that the scoring instrument was useful in rating candidates’ performance. Importantly, the OSCE scores distinguished between novice-expert learners meaning that the difficulty of the content was set at the appropriate level. The reliability of the results was adequate for a low-stakes formative exam. As we move towards CBME, a novel OSCE that addresses these important clinical scenarios would be important to include within a programme of assessment. This type of exam could also have potential utility as a high-stakes exam for qualification at the end of E&M training as it assesses unique scenarios that are expected of an Endocrinologist.

## Data Availability

The datasets used and/or analysed during the current study are available from the corresponding author on reasonable request.

## Abbreviations

ANOVA: Analysis of Variance
BRM: Borderline Regression Method
CBME: Competency-Based Medical Education
E&M: Endocrinology & Metabolism
EPA: Entrustable Professional Activities
GRS: Global Rating Scale
G-theory: Generalizability Theory
IM: Internal Medicine
ITC: Item-Total Correlation
OSCE: Objective Structured Clinical Examination
PD: Program Director
PGY: Post Graduate Year
SP: Standardized Patient
UofO: University of Ottawa

## References

[1] Frank JR, Snell LS, Cate O, Ten, Holmboe ES, Carraccio C, Swing SR (2010). Competency-based medical education: Theory to practice. Med Teach.

[2] Khan KZ, Ramachandran S, Gaunt K, Pushkar P (2013). The Objective Structured Clinical Examination (OSCE): AMEE Guide No. 81. Part I: An historical and theoretical perspective. Med Teach.

[3] Lockyer J, Carraccio C, Chan MK, Hart D, Smee S, Touchie C (2017). Core principles of assessment in competency-based medical education. Med Teach.

[4] Pugh D, Touchie C, Wood TJ, Humphrey-Murto S (2014). Progress testing: Is there a role for the OSCE?. Med Educ.

[5] Schuwirth LWT, Van der Vleuten CPM (2011). Programmatic assessment: From assessment of learning to assessment for learning. Med Teach.

[6] Van Der Vleuten C, Schuwirth LWT, Driessen EW, Dijkstra J, Tigelaar D, Baartman LKJ (2012). A model for programmatic assessment fit for purpose. Med Teach.

[7] Cook DA, Beckman TJ. Current concepts in validity and reliability for psychometric instruments: Theory and application. Am J Med. 2006;119(2):166. .e7-166.e16 . 10.1016/j.amjmed.2005.10.036 16443422

[8] Daniels V, Pugh D (2018). Twelve tips for developing an OSCE that measures what you want. Med Teach.

[9] Kane MT (1992). The Assessment of Professional Competence. Eval Health Prof.

[10] Messick S. Foundations in Validity: Meanings and Consequences in Psychological Assessment. New Jersey: Educational Testing Service; 1993. 2–12 p.

[11] Cook DA (2014). When I say… validity. Med Educ.

[12] Cook DA, Brydges R, Ginsburg S, Hatala R (2015). A contemporary approach to validity arguments: a practical guide to Kane ’ s framework. Med Educ.

[13] Hatala R, Cook DA, Brydges R, Hawkins R (2015). Constructing a validity argument for the Objective Structured Assessment of Technical Skills (OSATS): a systematic review of validity evidence. Adv Heal Sci Educ.

[14] Kane MT (2013). Validating the Interpretations and Uses of Test Scores. J Educ Meas.

[15] Tavares W, Brydges R, Myre P, Prpic J, Turner L, Yelle R (2017). Applying Kane’s validity framework to a simulation based assessment of clinical competence. Adv Heal Sci Educ.

[16] Howley L, Szauter K, Perkowski L, Clifton M, Mcnaughton N. Medical education in review Quality of standardised patient research reports in the medical education literature: review and recommendations. 2008;350–8. 10.1111/j.1365-2923.2007.02999.x 18298448

[17] Wood TJ, Humphrey-Murto SM, Norman GR (2006). Standard setting in a small scale OSCE: A comparison of the modified borderline-group method and the borderline regression method. Adv Heal Sci Educ.

[18] Downing SM (2004). Reliability: on the reproducibility of assessment data. Med Educ.

[19] Dagnone JD, Hall AK, Sebok-syer S, Klinger D, Davison C, Ross J (2016). Competency-based simulation assessment of resuscitation skills in emergency medicine postgraduate trainees – a Canadian multi-centred study. Can Med Educ J.

[20] Michelson JD, Manning L (2008). Competency assessment in simulation-based procedural education. Am J Surg.

[21] Chiu M, Tarshis J, Antoniou A, Bosma TL, Burjorjee JE, Cowie N (2016). Simulation-based assessment of anesthesiology residents’ competence: development and implementation of the Canadian National Anesthesiology Simulation Curriculum (CanNASC). Can J Anesth Can d’anesthésie.

[22] Naik VN, Brien SE (2013). Review article: Simulation : a means to address and improve patient safety. Can J Anesth.

[23] Royal College of Physicians and Surgeons of Canada. Objectives of Training in the Subspecialty of Endocrinology and Metabolism [Internet]. 2013. Available from: http://www.royalcollege.ca/rcsite/ibd-search-e?N=10000033+10000034+4294967098.

[24] Govaerts M, van der Vleuten CP (2013). Validity in work-based assessment: Expanding our horizons. Med Educ.

[25] Pugh D, Desjardins I, Eva K (2018). How do formative objective structured clinical examinations drive learning? Analysis of residents’ perceptions. Med Teach.

